# The evolution of dispersal conditioned on migration status

**DOI:** 10.1002/ece3.99

**Published:** 2012-04

**Authors:** Sarder Mohammed Asaduzzaman, Geoff Wild

**Affiliations:** Department of Applied Mathematics, The University of Western OntarioLondon, ON, N6A 5B7, Canada

**Keywords:** Kin selection, mathematical model, simulation, social evolution

## Abstract

We consider a model for the evolution of dispersal of offspring. Dispersal is treated as a parental trait that is expressed conditional upon a parent’s own “migration status,” that is, whether a parent, itself, is native or nonnative to the area in which it breeds. We compare the evolution of this kind of conditional dispersal to the evolution of unconditional dispersal, in order to determine the extent to which the former changes predictions about population-wide levels of dispersal. We use numerical simulations of an inclusive-fitness model, and individual-based simulations to predict population-average dispersal rates for the case in which dispersal based on migration status occurs. When our model predictions are compared to predictions that neglect conditional dispersal, observed differences between rates are only slight, and never exceed 0.06. While the effect of dispersal conditioned upon migration status could be detected in a carefully designed experiment, we argue that less-than-ideal experimental conditions, and factors such as dispersal conditioned on sex are likely to play a larger role that the type of conditional dispersal studied here.

## Introduction

Adaptive social behavior balances the selfish interests of an actor against those of genetically related neighbors (Hamilton 1964; Frank 1998). Information about the degree of relatedness between an actor and its neighbors can tip this balance, and sets the stage for the evolution of social behaviors with conditional expression.

Expression of behavior conditional upon the degree of relatedness between an actor and its neighbors appears to be widespread in nature. Greenbeards, for example, are genes that recognize copies of themselves in other individuals, then use this information to guide the conditional expression of the social behavior of their bearer (Gardner and West 2009). Though the concept was originally a hypothetical one (Hamilton 1964; Dawkins 1976), greenbeard genes have been found in the red fire ant, *Solenopsis invicta* (Grafen 1998; Keller and Ross 1998), and greenbeard-like behaviors have also been identified in a number of taxa, ranging from the slime mold, *Dictyostelium discoideum* (Queller et al. 2003) to side-blotched lizards, *Uta stansburiana* (Sinervo et al. 2006).

The “imprinting” of genes expressed in the placenta and brains of mammals constitutes yet another example of conditional social behavior (again, perpetrated by genes). Imprinted genes are expressed differently depending upon whether they are maternally or paternally inherited. Conditional expression, here, is maintained, because parents are related by differing amounts to the social partners of their offspring (Haig 2000). Imprinting, or rather the breakdown of imprinting, has been implicated in certain genetic disorders of humans (Úbeda 2008), and may contribute to abnormal psychosocial development (Badcock and Crespi 2008).

As the discussion above suggests, theoretical work on conditional expression of social behavior is varied. Moreover, one could argue that the basic conclusions drawn by this body of work are, in a sense, mixed. In some cases, theoretical investigations carried out under the assumption of conditional expression of behavior have led to predictions that differ markedly from those obtained by investigations carried out under the assumption of unconditional expression. El Mouden and Gardner (2008) have shown that costly expression of helpful behaviors can be advantageous when expression is conditional on “migration status” (i.e., on whether one is native or nonnative to the place in which it breeds), a result that is not only quite different from that obtained in the absence of such conditioning (Taylor 1992; Wilson et al. 1992), but also a result that matches more closely with the observation that altruism and cooperation are widespread in nature. In other cases, assumptions about conditional expression do not change the standard model predictions. Wild and West (2009), for example, have shown that imprinting of genes responsible for sex-allocation behavior changes corresponding unconditional predictions about the sex ratio only very slightly (for certain mating systems). In cases like these, giving consideration to conditional expression of social behavior does not appear to sharpen our understanding of nature at all.

Given the mixed conclusions, then, it seems reasonable to question the general significance of conditional expression: should we readily expect that conditional expression of social behavior results in an improved match between theory and observations? We will address this question, at least in part, by comparing the predictions of a models for the evolution of conditional expression of natal dispersal behavior to those made by a model for the evolution of its unconditional counterpart.

Natal dispersal occurs when an individual leaves its birthplace to mate and reproduce elsewhere. Although, much theoretical work has focused on conditional dispersal (e.g., Crespi and Taylor 1990; Ronce et al. 1998, 2000; Travis et al. 1999; Kisdi 2004; Bonte and Pena 2009), dispersal conditioned upon relatedness cues has been relatively neglected. Although there is some evidence to suggest that organisms can adjust their behavior in response to migration-status cues (Taylor and Crespi 1994), it remains unclear if such adjustments would significantly alter predictions about the evolution of natal dispersal.

**Figure 1:**
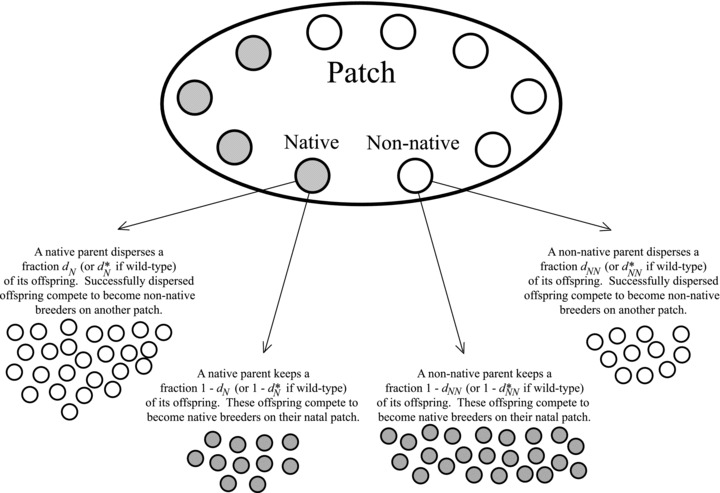
We model the evolution of offspring dispersal rate when this rate is determined by a parent (an adult). In our model, an adult that breeds on its natal patch (a “native,” represented by a gray circle) disperses some fraction of its offspring, denoted *d*_*N*_ or 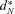. An adult that breeds on a patch other than its natal patch (a “non-native,” represented by a white circle) also disperses a fraction of its offspring, but that fraction is denoted 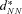 or *d*_*NN*_. The dispersal rates 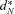 (or *d*_*N*_) and 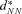 (or *d*_*NN*_) can be different, and so we have conditional expression of dispersal phenotypes—expression conditional upon a parent’s own “migration status.”

In this paper, we build an inclusive-fitness model for the evolution of natal dispersal when that dispersal is under parental control. In other words, we investigate how the fraction of offspring dispersed by a parent changes in response to evolutionary forces (primarily selection). We build on the work of El Mouden and Gardner (2008) and consider dispersal expressed conditional upon a parent’s own migration status (Fig. 1). In particular, we look to compare the evolution of dispersal conditioned on migration status with the evolution of its unconditional counterpart. Overall, our goal is to determine the extent to which this kind of conditional behavior changes predictions about population-average levels of dispersal.

We determine the stable dispersal strategies for native and nonnative parents, respectively, using numerical investigation of our model. We also investigate the long-term evolution of dispersal conditioned on migration status using individual-based simulation. With the exception of a few (relatively extreme) cases, the agreement between inclusive-fitness predictions and simulation results is good. When we compare predictions about the evolution of conditional dispersal to benchmark predictions made by “classical” unconditional models (Taylor 1988; see below), we find only slight differences. Overall, we conclude that such differences are unlikely to be noticed in the field.

## A Benchmark Model of Unconditional Dispersal

In this section, we review the model for the evolution of unconditional dispersal presented by Taylor (1988), as well as the predictions that this model makes.

Consider a haploid, asexual population undergoing discrete, nonoverlapping generations (Taylor considered sexual diploid and haplodiploid systems, but his model can be applied to haploid, asexual organisms as well). We assume that the population is arranged into a very large number of patches. Each patch is assumed to be identical, and each is assumed to support exactly *N* breeding adults.

Given the assumptions above, it is easy to show that a parent who disperses a slightly greater-than-average proportion of its offspring enjoys a selective advantage whenever 

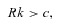
1
where *R* is the relatedness between the focal parent and the average offspring born on its patch, *k* represents the benefit dispersal confers on those individuals who do not disperse, and *c* represents the marginal fitness cost of dispersing. Readers familiar with social evolutionary theory will notice that (1) is analogous to Hamilton’s famous rule for the selective advantage of altruism.

In broader terms, the sign of *Rk*–*c* determines the sign of the selection gradient acting on the population-average dispersal rate d*. When *Rk*–*c* > 0, selection acts to increase d*, when *Rk*–*c* < 0, selection acts to decrease d*. When *Rk*=*c*, the population-average dispersal rate is at evolutionary equilibrium. Such equilibria can be identified by substituting *k*= (1 –d*)/(1 –cd*) and *R*= 1/(*N*– (*N*– 1)*k*^2^) into *Rk*–*c*, then solving for d*. Carrying out this procedure, one finds

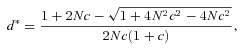
2
which also happens to be stable against population-wide perturbations (i.e., convergence stable; Christiansen, 1991). Equation (2) will serve as a benchmark against which we will compare the effect of conditional dispersal.

## A Model of Dispersal Conditioned on Migration Status

In this section, we set out most of the basic assumptions used in our model for the evolution of dispersal conditioned on migration status. For the reader’s convenience, a brief description of all notation introduced in the main text is given in Table 1.

**Table 1:** Summary of notation used in the text.

Symbol	Explanation
α_*ij*_	The native component of the *i*-fitness of a native mutant breeding on a type-*j* patch
β_*ij*_	The native component of the *i*-fitness of a nonnative mutant breeding on a type-*j* patch
β_*ij*_	The nonnative component of the *i*-fitness of a native mutant breeding on a type-*j* patch
δ_*ij*_	The nonnative component of the *i*-fitness of a nonnative mutant breeding on a type-*j* patch
*c*	Cost of dispersal
*d*_*N*_	Mutant native dispersal rate
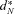	Wild-type native dispersal rate
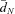	Average dispersal rate on a patch that supports at least one mutant native
*d*_*NN*_	Mutant nonnative dispersal rate
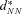	Wild-type nonnative dispersal rate
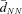	Average dispersal rate on a patch that supports at least one mutant nonnative
〈d^*^〉	Stable population-wide average dispersal rate, when dispersal is based on migration status
*K*	Number of offspring produced by a each adult
*M*	Number of patches (ideally infinite in the kin selection model)
*N*	Patch size
*p*_*j*_(*x*, *y*)	Probability that a site on a patch that had supported *j* natives is won by a native in the next generation, given the local natives and nonnatives dispersed offspring at rate *x* and *y*, respectively
*P*_*ij*_(*x*, *y*)	Probability that a patch that had supported *j* natives will support *i* natives in the next generation, given the local natives and nonnatives dispersed offspring at rate *x* and *y*, respectively
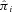	Frequency of type-*i* patches at demographic equilibrium
*R*_*j*_	Relatedness between a native adult and the average native adult breeding on the same patch (includes relatedness to self)
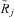	Relatedness between a nonnative adult and the average nonnative adult breeding on the same patch (includes relatedness to self)
**u**_*j*_	Vector that stores the relative abundance of natives and nonnatives, respectively, breeding on a type-*j* patch at demographic equilibrium
**v**_*i*_	Vector that stores the reproductive value of a native individual and a nonnative individual, respectively, breeding on a type-*i* patch
**W**_*ij*_	Matrix-valued function that stores α_*ij*_, β_*ij*_, γ_*ij*_, and δ_*ij*_, respectively
W^*^	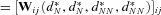
Δ*W*^*N*^	The inclusive-fitness effect of increased native dispersal
Δ*W*^*NN*^	The inclusive-fitness effect of increased nonnative dispersal

### Preliminary details

Like Taylor (1988), we consider a population with discrete, nonoverlapping generations that consists of a very large number of habitat patches (*M*) of identical quality where each patch supports exactly *N* individuals (i.e., *N* breeding sites per patch). Unlike Taylor (1988), however, we assume that individuals in the population are haploid and asexual. This assumption leads to more straightforward calculations.

To keep track of migration status, we classify each patch according to the number of native breeders (adults born on that patch) it supports. We use *j*= 0, 1, …, *N* to indicate the number of natives breeding on that patch. Thus, on a type-*j* patch, there are *j* natives, and (*N*–*j*) nonnatives. The frequency of a type-*j* patch, denoted by π_*j*_, is expected to fluctuate over time. To indicate that these frequencies have reached a demographic equilibrium (but not necessarily evolutionary equilibrium), we furnish π_*j*_ with a hat and write the distribution of patch types as 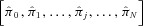.

### Phenotypes

As mentioned above, the phenotypes of interest relate to the fraction of offspring dispersed by a parent. We consider the evolution of two such phenotypes: (1) a phenotype that is expressed only by an individual breeding on its natal patch (native dispersal rate), and (2) a phenotype that is expressed only by an individual breeding away from its natal patch (nonnative dispersal rate). We assume that every individual possesses genes for both dispersal phenotypes, though only one phenotype is ever expressed (genes for the other phenotype are silent). Our immediate goal is to evaluate the success of a rare mutant form of one or the other conditional phenotypes in a wild-type (i.e., nonmutant) population at demographic equilibrium. Mutations are assumed to be rare, and so we neglect the possibility of double mutants.

### The model life cycle

We will assume that the overall population dynamics are determined by a series of discrete life-cycle events that occur in the same order in every generation. Each of these events (a–c) is described (in order) below.

(a) *Birth—*During the first event of the life cycle, adults produce offspring. We use *K* to denote the very large number of offspring produced by each adult (natives and nonnatives, mutants and wild types). On a type-*j* patch, then, *Kj* offspring are produced by native adults, and *K*(*N*–*j*) offspring are produced by nonnatives.

(b) *Dispersal—*In the second phase, each adult disperses a certain fraction of its brood. We use 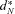 and 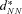 to denote the fraction of offspring dispersed by wild-type native and nonnative parents, respectively. Thus, on a type-*j* patch that supports only wild-type individuals, we find a total of

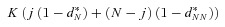
3
offspring that do not disperse. Rather than disperse these offspring remain on their natal patch and compete as natives once dispersal is complete (Fig. 1). Note that the 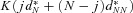 other offspring produced on the type-*j* patch in question disperse to (possibly) compete as nonnatives elsewhere (Fig. 1).

We use 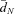 and 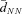 to denote, respectively, the average native phenotype found on a patch that supports at least one native mutant, and the average nonnative phenotype found on a patch that supports at least one nonnative mutant. Thus, on a type-*j* patch that supports at least one native mutant, we expect to find

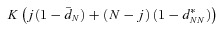
4
native offspring once dispersal is complete. Similarly, once dispersal is complete, we expect to find

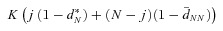
5
native offspring on a type-*j* patch that supports at least one nonnative mutant. Note that the since mutations are globally rare, we can neglect the possibility that both native and nonnative mutants occur together on the same patch.

We allow for the possibility that dispersal is costly. Specifically, we assume that a fraction, *c*, of dispersed offspring never find a new patch, and perish as a result. The remaining fraction (1 –*c*), however, do find a new patch. If each patch receives an equal share of successful dispersers, then the number of nonnative offspring found on any patch once dispersal is complete is given by 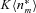 where

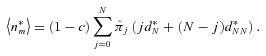
6
The rareness of mutations allows us to use the previous expression to describe immigration to all patches, regardless of whether they had previously supported mutant breeders or not.

(c) *Competition—*We assume that, by the end of the dispersal phase of the life cycle, all adults have perished leaving *N* breeding sites per patch vacant. Competition for vacant breeding sites on a given patch then occurs at random among the native and nonnative offspring found there (technically, uniformly at random, with replacement). For convenience, we define

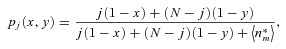
7
and we note that 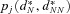 gives the probability that a breeding site on a patch that had supported only wild-type individuals will be won by an offspring that is native to that patch. Similarly, 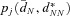 gives the probability that a breeding site on a patch that supported at least one native mutant will be won by an offspring that is native to that patch, and 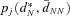 gives the probability that a breeding site on a patch that supported at least one nonnative mutant will be won by an offspring that is native to that patch.

If we now define 

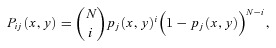
8
then we can use this expression to describe the probability that type-*j* patch becomes a type-*i* in the next generation (the transition probabilities). For a patch that currently supports only wild-type individuals, we have 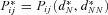. Similarly, 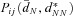 describes the transition probabilities for a patch that supports at least one native mutant, while 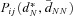 describes those for a patch that supports at least one nonnative mutant. Wild-type transition probabilities determine the distribution of patch types at demographic equilibrium, according to the equations

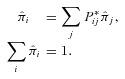
9
Mutant transition probabilities are used to determine mutant fitness, and it is mutant fitness to which we turn our attention now.

### Mutant fitness

We define the *i*-fitness of an individual as the number of its offspring found breeding on a type-*i* patch one generation into future. An individual’s *i*-fitness has both native and nonnative components. The native component counts the number of offspring breeding as natives on type-*i* patch, and the nonnative component counts the number of offspring breeding as nonnatives on a type-*i* patch.

The fitness of a mutant will depend on whether it is native or nonnative to the patch on which it breeds. A native mutant disperses a fraction *d*_*N*_ of its offspring; when this mutant breeds on a type-*j* patch, we use 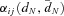 and γ_*ij*_(*d*_*n*_) to denote the native and nonnative components of its *i*-fitness, respectively. A nonnative mutant disperses a fraction *d*_*NN*_ of its offspring; and when this mutant breeds on a type-*j* patch, we use 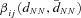 and δ_*ij*_(*d*_*NN*_) to denote the native and nonnative components of its *i*-fitness, respectively.

Using the description of the life cycle provided above, one can determine that 

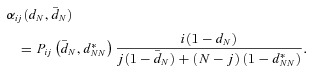
10

We see that α_*ij*_ is the product of two terms. The first term, 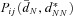, is the probability that the patch that currently supports *j* natives will support *i* natives in the next generation. The second term is the number of next-generation native spots expected to be won by the focal mutant’s own offspring. In words, then, α_*ij*_ is the number of mutant offspring that compete successfully on the focal mutant’s patch, conditional upon there being exactly *i* breeding spots reserved for locally produced offspring.

The model life cycle also tells us that 

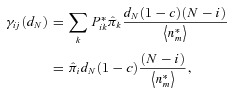
11
where the second equality follows from equation (9). In this case, *d*_*N*_(1 –*c*) is the probability that a given mutant offspring disperses successfully. Given that the offspring dispersed successfully, 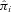 represents the probability that it found a patch that ultimately supported (*N*–*i*) nonnative breeders. Lastly, 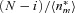 represents the probability that the particular offspring being considered wins one of the (*N*–*i*) nonnative spots that are being contested by 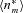 other immigrants.

The remaining fitness functions are 

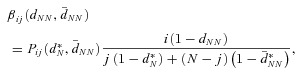
12
and

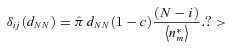
13
Equations (12) and (13) are derived and interpreted in the same way as their counterparts (10) and (11) above.

### Inclusive-fitness effects

We store the fitness functions above in the matrix-valued function, 

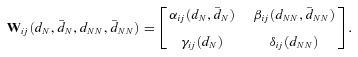
14
Under the assumption of weak selection, Taylor and Frank (1996) have shown that the fate of a mutant allele that alters either native or nonnative dispersal can be determined using the dominant left and right eigenvectors of the matrix

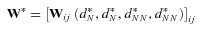
15
(call these eigenvectors **v**= [ **v**_*i*_]_*i*_ and **u**= [ **u**_*j*_]_*j*_, respectively), and marginal fitness components expressed using partial derivatives of the elements of the matrix 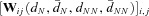. Following the approach of Taylor and Frank (1996), we focus on two expressions:

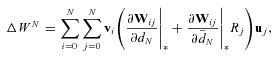
16

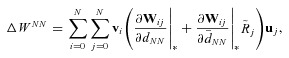
17
where “|_*_” indicates that partial derivatives are to be evaluated by setting all dispersal rates equal to their corresponding wild-type values. The symbol *R*_*j*_ in (16) denotes the relatedness between a native and the average native breeding on the same type-*j* patch. The symbol 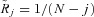 for (*j*≤*N*– 1) in (17) denotes the relatedness between a nonnative and the average nonnative breeding on the same type-*j* patch. Relatedness coefficients are calculated in Appendix A1. Note that both coefficients account for relatedness to self.

The expression in (16) predicts the fate of a mutant lineage that alters its native dispersal rate, and so we call this expression “the inclusive fitness effect of native dispersal.” The expression in (17) predicts the fate of a mutant lineage that alters its nonnative dispersal rate, and so we call this expression “the inclusive fitness effect of non-native dispersal.” When Δ*W*^*N*^ (resp. Δ*W*^*NN*^) is greater than zero, a native (resp. non-native) mutant that disperses slightly more offspring than the native (resp. nonnative) wild type will invade; in other words, selection favors an increase in 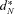 (resp. 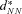). When Δ*W*^*N*^ (resp. Δ*W*^*NN*^) is less than zero, a native (resp. nonnative) mutant that disperses slightly fewer offspring than native (resp. nonnative) wild type will invade; in other words, selection favors a decrease in 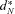 (resp. 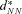). Conditional dispersal rates are at evolutionary equilibrium if Δ*W*^*N*^ and Δ*W*^*NN*^ are zero. An evolutionary equilibrium will be considered stable when it is the long-term result of a selective process. With this loose definition of stability, nonequilibrium wild-type dispersal rates (i.e., boundary rates, of zero or one) might also be stable.

## Methods of Analysis

### Numerical procedure to find stable dispersal rates

Because our model is not analytically tractable, we used it to simulate the evolution of 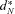 and 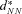 numerically. The result of this numerical simulation is what we call a stable phenotype pair.

Our numerical simulation started with an initial guess of stable value of 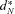 and 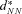 based on fixed values of *N* and *c*. Using this guess, the patch distribution at equilibrium was determined by solving (9). The equilibrium distribution of patch types, in turn, allows the signs of the inclusive-fitness effects Δ*W*^*N*^ and Δ*W*^*NN*^ to be determined using equations (16) and (17), respectively.

Based on the sign of Δ*W*^*N*^ and Δ*W*^*NN*^, our initial guess for stable dispersal rates could be improved. If Δ*W*^*N*^ (resp. Δ*W*^*NN*^) was positive, 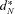 (resp. 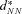) was increased by a small amount; if Δ*W*^*N*^ (resp. Δ*W*^*NN*^) was negative, 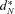 (resp. 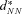) was decreased by a small amount. The new guesses were then used to recompute Δ*W*^*N*^ and Δ*W*^*NN*^ and further refinements were made in the same way until either the size of the inclusive-fitness effects fell below some preset threshold, or the boundary of the phenotype space (zero or one) was reached.

For given *N* and *c*, the numerically determined, stable phenotype pair was used to compute the population-average dispersal rate, 〈d*〉, according to the equation

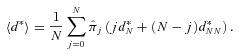
18

It is 〈d*〉 that we compare to Taylor’s (1988) result in equation (2).

### Individual-based simulation

To validate the numerical approach described above, we devised an individual-based simulation. Because simulations were computationally expensive, only a limited number of parameter combinations were investigated. We investigated for *N*= 2, 4, 6, 8, and *c*= 0.1, 0.2, … , 0.9. We simulated a population made up of *M*= 200 patches, and so at any time during the simulation each of *MN* positions was occupied by an individual.

A typical simulation began by furnishing the individuals at each of *MN* positions with randomly determined pairs of dispersal phenotypes (one phenotype to be expressed when native, and the other to be expressed when nonnative), and a randomly determined migration status. The dispersal phenotype pair and migration status at each position was updated by simulating the birth, dispersal, and competition events described above. A small mutation rate was added to the simulation to ensure it explored a greater fraction of the phenotype space. Complete MATLAB scripts are presented in Appendices A2 and A3. Preliminary investigations suggested that 5000 simulated generations provided more than enough time for stable dispersal rates to become established. Thus, all simulations were stopped after 5000 generations.

When the stable dispersal rate of nonnatives was predicted to be zero by the numerical investigation, we simulated only the dispersal rate of natives. In this case, we fixed the nonnative dispersal rate to zero level to reduce variation (noise). All other aspects of the simulation were unchanged.

For each parameter combination (fixed *N* and *c*), we devised an estimate of the stable conditional dispersal rates (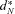 or 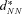) based on 50 replicate simulations. Specifically, an average evolutionary trajectory was determined for 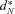 and 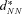 by calculating the appropriate mean for the collection of 50 replicates during every generation. Each average trajectory was then time-averaged over the last 2000 generations to arrive at the estimate for stable 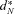 and 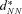, respectively. Ninety-five percent confidence intervals for our estimates were based standard errors of trajectories time-averaged over the last 2000 simulated generations.

## Results

### Summary of numerical results

**Figure 2:**
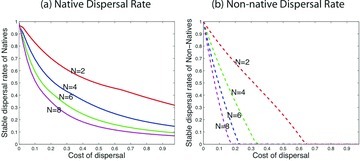
The relationship between the stable conditional dispersal rates 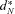 (panel a) and 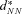 (panel b), and the model parameters *c* (cost of dispersal) and *N* (patch size).

Our model predicts that stable levels of conditional dispersal will decrease with increasing *c*, and with increasing *N* (Fig. 2). Similar predictions are made by standard models for the evolution of unconditional dispersal (e.g., Taylor 1998). All else being equal, an increase in *c* increases the inclusive-fitness cost of dispersing one’s offspring—the decreased stable levels of dispersal are simply a response to this disincentive. Along the same lines, increased *N* effectively dilutes the inclusive-fitness benefit of dispersal, again resulting in decreased stable dispersal rates.

**Figure 3:**
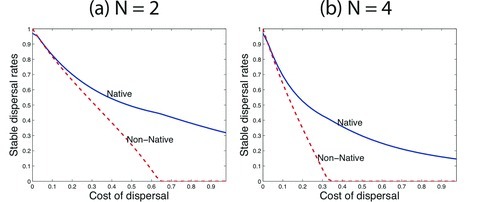
The relationship between the stable conditional dispersal rates 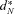 and *d*_*NN*_ for varying *c* (cost of dispersal) when (a) *N*= 2, and (b) *N*= 4 (*N* is patch size).

In addition to the consistent relationships between dispersal rates and model parameters, we have observed a consistent relationship between the dispersal rates themselves. Specifically, we found that the stable rate of dispersal of natives (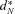) is always greater than that of nonnatives (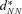) (Fig. 3). Certainly, natives are more closely related to the offspring produced on their patch than are their nonnative neighbors. Inclusive-fitness benefits of dispersal, therefore, accrue at a higher rate for natives, and the relatively higher native dispersal rates we have found represent a response to this extra incentive.

### The match between numerical and individual-based simulations

**Figure 4:**
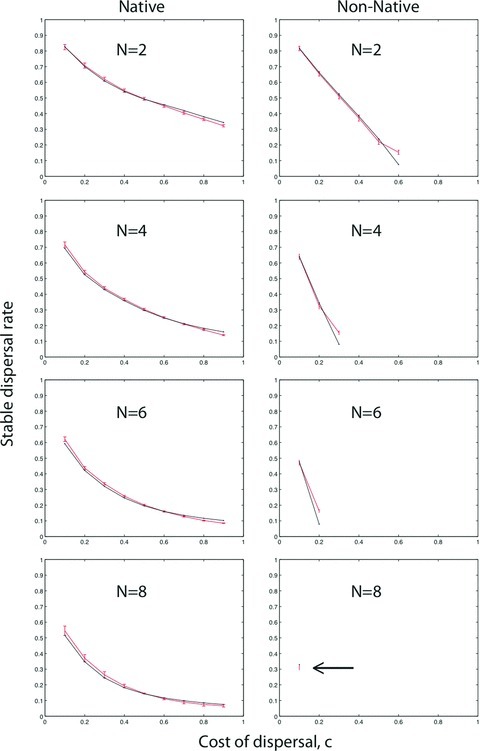
Comparison between predictions about stable, conditional dispersal rates generated by numerical simulation (black lines) and those generated by individual-based simulation (red lines) for varying *c* (cost of dispersal) and *N* (patch size). Ninety-five percent confidence intervals accompany individual-based simulation results. Results for nonnative dispersal rates (right panels) are truncated when numerical simulation predicts 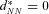. Note that this means there is only one observation for the *N*= 8 panel.

We found a qualitative agreement between numerical and simulation results, but the level of quantitative agreement was mixed and depended on parameter combinations considered (Fig. 4).

In many cases, the individual-based simulation tended to agree with the numerical results, in the sense that the 95% confidence interval over the last 2000 generations captured the numerical result (Fig. 4). When the 95% confidence interval did not contain the numerical prediction, the extent of the disagreement was, typically, only very slight (Fig. 4). On the whole, the agreement between numerical and individual-based simulations was good.

We should point out that in a minority of cases, the quantitative agreement between numerical and individual-based simulation was quite pronounced. These larger disagreements occurred for larger values of *c* and *N*—where numerical predictions for 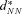 fell below 0.1 (Fig. 4, right panels). Larger discrepancies involving 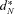 were eliminated in those cases where numerical results predicted 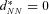 (i.e., in those cases where our individual-based simulation procedure set 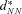 equal to zero), and so low nonnative dispersal rates appear to be the driving force behind the disagreements, when they occur. One factor contributing to the larger disagreements could be the stochastic effects associated with the small size of the subpopulation nonnative at larger *N* and *c*, but this is conjecture.

### The effect of conditioning on the population-average dispersal rate

Our main goal was to compare the population-average dispersal rate under conditional dispersal (〈d*〉) with the unconditional result given by Taylor (1988). Because there is some disagreement between certain numerical and individual-based simulation results, we calculated 〈d*〉 using numerical results (eq. 18) and using data from the individual-based simulation.

**Figure 5:**
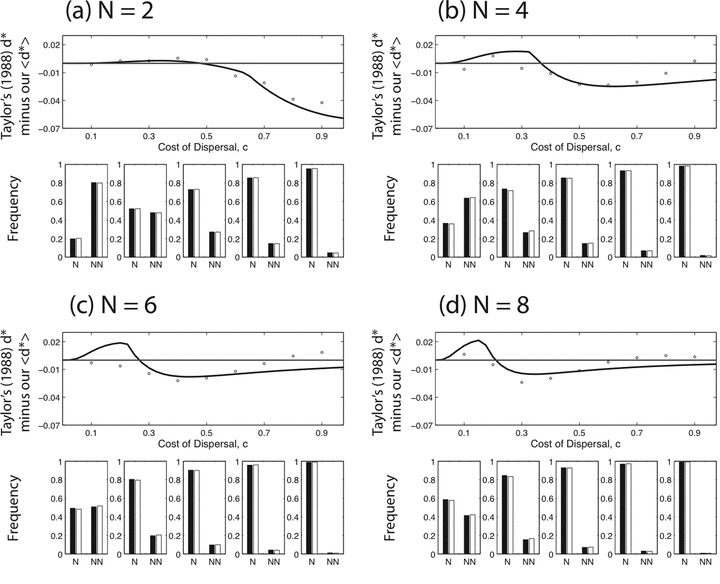
Comparisons between Taylor’s (1988) predicted dispersal rate, d* (eq. 2) and the population-average dispersal rate 〈d*〉 predicted by our model with dispersal conditioned on migration status. We have plotted the difference between Taylor’s predictions and numerical simulation results (solid curve) as well as the difference between Taylor’s predictions and individual-based simulation results (open circles), for various values of *c* and *N*. In all cases, the comparison showed that our predictions differ from those of Taylor by only a small amount. Below each comparison, we also present the frequency of natives (*N*) and nonnatives (*NN*) for numerical (solid bars) and individual-based simulations (open bars) for *c*= 0.1, 0.3, 0.5, 0.7, 0.9) (left to right).

At lower costs of dispersal, Taylor’s (1988) population-average dispersal rate is greater than that predicted by our numerical model, and at higher costs Taylor’s (1988) population-average dispersal rate is less than that predicted by our numerical model (Fig. 5). The switch between the two cases (Taylor’s prediction greater than those of our numerical model on one hand, and Taylor’s predictions less than those of our numerical model on the other) occurs at an intermediate cost that, itself, decreases with decreasing *N* (Fig. 5).

Comparison between Taylor’s predictions and data from individual-based simulation reveals a pattern that is similar to the one revealed by the numerical results, though discrepancies occur in those cases where 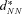 is small (as discussed above). In some cases, Taylor’s predicted population-average dispersal rate lies between population-average predictions from our numerical and individual-based simulations, respectively. That said, the absolute difference among any of the predicted population-average dispersal rates (Taylor’s predictions, our numerical predictions, our predictions from individual-based simulations) is small (note the vertical scales in Fig. 5). In fact, as we argue below, the small differences between Taylor’s predictions and any of our predictions (the largest such difference is approximately 0.06; see Fig. 5) suggest to us that dispersal conditioned upon a parent’s migration status is unlikely to confound match between theory and data.

## Discussion

In the model we develop above, an adult that has dispersed does not breed alongside relatives, and so—relative to one that has not dispersed—that adult can afford to disperse its own offspring in a more selfish manner. Using migration status as a cue for conditioning the dispersal of one’s offspring, then, will confer a clear selective advantage. Still, most theoretical predictions are generated under the assumption that such conditional behavior does not occur. Since there is empirical evidence to suggest that phenotypic expression can depend on a variety of kin-recognition cues (e.g., Breed and Julian 1992; Sharp et al. 2005), including an individual’s migration status (sex-ratio data from *Hoplothrips pedicularius* collected by Taylor and Crespi [1994] suggests that this is so), one might naively expect the predictions of standard theory to be incorrect. Our goal was to assess the extent to which dispersal conditioned upon migration status might confound the match between standard theory and observations. We chose the model of unconditional dispersal presented by Taylor (1988) as a benchmark against which to compare our model.

We observed quantitative differences between Taylor’s (1988) predictions and the population-average dispersal rates predicted by our model that typically ranged between 0 and 0.01 in absolute value (but in one case the difference was as high as 0.06). That said, it is not immediately clear that these differences would, in practice, cause problems for the match between theory and data.

Suppose, for the sake of argument, that one was able to overcome the difficulties associated with determining parameter values (e.g., see Wolff 1994). In that case, rough calculation suggests that a sample of size between 100 and 10, 000 would be needed to detect the differences between our predictions and those of Taylor (1988) with 95% confidence (e.g., Montgomery et al. 2011, p. 212). A sample size on the order of 100 is not unheard of in field investigations and experiments (e.g., Greenwood et al. 1978; Harvey et al. 1979), and so the effect of conditional dispersal could potentially be detected in a carefully designed experiment. That said, field and experimental conditions are seldom perfect. Microhabitat variation, for example, has been found to alter passive dispersal by black-fly neonates significantly (Fonseca and Hart 1996). This “noise” introduced by variation of this kind could have the potential to obscure the detection of even six dispersers of 100 offspring—the upper end of the range of values quoted above.

Other adaptive changes to the dispersal rate provide additional challenges for the potential match between dispersal theory and data. Theoretical work has demonstrated the advantage of conditioning dispersal based on, among other things, habitat quality (Greenwood-Lee and Taylor 2001; Leturque and Rousset 2002), sex and the sex ratio (Leturque and Rousset 2003; Wild and Taylor 2004), and brood size (Kisdi 2004). Although we cannot readily compare the work cited here to the results of Taylor (1988), it seems reasonable to suggest that it would be very difficult to disentangle the predictions made by this work from the subtle effect of conditioning based on migration status. Overall, then, we conclude that ignoring the possibility that parents condition dispersal of offspring based on their own migration status is not likely to cause a problem for the match between dispersal theory and data.

Our conclusion that information about migration status does little to change the overall level of dispersal in a population is particularly interesting in light of previous work. As mentioned in the introduction, El Mouden and Gardner (2008) found that when helping (and indeed harming) can be expressed conditional on migration status, natives help and nonnatives do not help. Their predictions are quite different from the predictions of models that assume helping is not informed by migration status—those “unconditional” models predict that no individual helps (Taylor 1992; Wilson et al. 1992). In essence, then, El Mouden and Gardner (2008) show that information about migration status can increase the overall level of help in the population to a level that is in proportion to the number of native individuals in a populations; and as we see from Figure 5, the number of natives can be quite substantial. With that in mind, one might ask: why is it that migration status is of so little consequence to overall level of dispersal, when it has the potential to change overall levels of helping so substantially? Unfortunately, we can offer only a partial answer to this question.

In the model presented by El Mouden and Gardner (2008), patch demographics are not substantially affected by changes in individual behavior—a consequence of the assumption that fitness effects are additive and the assumption that selection is weak. Essentially, the demographic effects of helping/harming in El Mouden and Gardner (2008) are small, even when natives and nonnatives have very disparate phenotypes. We suggest that the minimal demographic sensitivity found in El Mouden and Gardner (2008) eliminates “higher order” effects of selection (e.g., substantial demographic change) that (possibly) work against population-wide evolutionary change. To clarify this point, consider the dispersal model we present here. Although selection in our model is weak, we observed substantial demographic change associated with changes in the evolving traits (Fig. 5). In particular, demographic changes seem to act to buffer the population-wide level of dispersal against the effects of relatively extreme conditional dispersal rates. For example, when nonnative dispersal rates are zero (at high cost of dispersal *c*), the population as a whole is dominated by native individuals that disperse offspring at a low, but nonzero, rate (Fig. 5). The demographic situation in this case is not far from one we expect to see in a model with unconditional dispersal (average dispersal rates in such a model are also low), and so the cost–benefit balancing made by individuals in the dominant native subpopulation closely follows that made by individuals who are unaware of their own migration status. In other words, noticeable demographic shifts appear to act against substantial population-level changes. We must emphasize that the effect of demography here is primarily speculative, and future work should address its consequences for conditional social behavior in greater detail.

## A1. Relatedness

In this appendix, we outline the calculation of *R*_*i*_ (for *i*≥ 1), the relatedness between the a native adult and the average native adult that breeds on its patch (this includes relatedness to self). We follow Taylor and Frank (1996) and determine the set of *R*_*i*_ values under the assumption of that selection is absent. A recursive argument shows that, in the long run, each *R*_*i*_ satisfies

(A1)

thus, the set of *R*_*i*_ values can be determined by solving this system of equations (this was done numerically).

## A2. Individual-based simulation of the joint evolution of conditional dispersal rates

In this section, we provide a version of the Matlab script used to execute an individual-based simulation of the joint evolution of  and .

## A3. Individual-based simulation of the evolution of native dispersal rate

In this appendix, we provide the Matlab script that executes an individual-based simulation of the evolution of *d*_*N*_ alone. This simulation was used when the numerical model predicted .
